# Editorial: Emerging contaminants and their effect on agricultural crops

**DOI:** 10.3389/fpls.2023.1296252

**Published:** 2023-10-24

**Authors:** M. Naeem, Ritu Gill, Sarvajeet Singh Gill, Kashmir Singh, Adriano Sofo, Narendra Tuteja

**Affiliations:** ^1^ Department of Botany, Aligarh Muslim University, Aligarh, India; ^2^ Centre for Biotechnology, Maharshi Dayanand University, Rohtak, India; ^3^ Department of Biotechnology, Panjab University, Chandigarh, India; ^4^ School of Agricultural, Forestry, Food and Environmental Sciences, University of Basilicata, Potenza, Italy; ^5^ Plant Molecular Biology Group, International Centre for Genetic Engineering and Biotechnology, New Delhi, India

**Keywords:** agricultural crops, climate change, climatic factors, emerging contaminants, nitrogen and phosphorus nutrient cycling, particularly polyethylene, polyvinyl chloride, *Salvia miltiorrhiza*

## Introduction

Agriculture is a primary source of food for global population, and just three staple cereals (rice, maize, and wheat) provide nearly half of the world’s food calories and also helps in ensuring global food security (Grote et al., 2021; Erenstein et al., 2022; FAO, 2023). Rapid increase in global population, frequently changing global climatic conditions, COVID-19 outbreak significantly challenged the food production and pose serious threat to food security. Over the years, urbanization, rapid industrialization, and luxurious use of agrochemicals in modern agriculture accumulated many new contaminants in agricultural system (Bayabil et al., 2022). Ever increasing global population and frequently changing global climatic conditions in the era of war or war like global situation pose excessive burden on global food production through the use of chemicals, high yielding crops and significant intensification of agricultural land (Galanakis, 2023). Emerging contaminants (ECs) in agriculture are substances that are not traditionally regulated but have been recently identified/recognized as potential environmental pollutants such as nano- and microplastics, Per- and polyfluoroalkyl substances (PFAS), endocrine-disrupting chemicals (EDCs), antibiotics, hormones, pain relievers etc and becoming a concern due to their potential adverse effects on crops, soil, water, and the environment. These contaminants can originate from various sources, including agricultural practices (use of agrochemicals, pesticides, and herbicides), industrial activities, personal care products, pharmaceuticals, and urban runoff. In the modern era, to boost agri-production, new formulations of pesticides/herbicides are continually developed to combat evolving pest and weed populations (Kilani-Morakchi et al., 2021; Rajak et al., 2023). Long term use of contaminated irrigation water, fertilizers, pesticides, and soil amendments leads to the accumulation of cadmium, lead, and arsenic in agricultural soils and affecting the plant ecosystem health significantly (Sun et al., 2021; Mohanty and Das, 2023; Rashid et al., 2023). Additionally, the use of pharmaceuticals and veterinary drugs in livestock farming can lead to the presence of residues in manure and runoff water, potentially impacting aquatic ecosystems and soil microorganisms, and the agricultural produce (Charuaud et al., 2019; Khan and Barros, 2023; Nightingale et al., 2023). Moreover, it has been realized that ECs like antibiotics, hormones and pain relievers have the potential to accumulate in crops, exerting notable effects on their growth, development, and ultimately, the diminished quality of the products. For example, specific antibiotic exposure has been found to disrupt the normal growth and development processes in plants (Zhang et al., 2017; Li et al., 2023). EDCs may enter agricultural systems through water sources or agricultural chemicals, affecting reproductive and developmental processes in crop plants and animals (Pridemore, 2021; Sharma et al., 2022; Thacharodi et al., 2023). PFAS compounds contain at least one aliphatic perfluorocarbon moiety and have also been detected in soil and water near agricultural areas due to anthropogenic activities, raising concerns about their impact on crops and the food chain (Adu et al., 2023; Karamat et al., 2023; Nassazzi et al., 2023). Frequent changes in global climate patterns and increased global trade have facilitated the spread of new and more virulent plant pathogens, such as bacteria and fungi, which have the potential to pose serious threat to crop production (Singh et al., 2023).

Nano- and microplastics can impact soil structure and potentially reduce water and nutrient retention in soil, affecting crop growth (Jia et al., 2023; Zantis et al., 2023). Additionally, microplastics can be accumulated by crops, raising concerns about food safety and human health (Iqbal et al., 2023; Quilliam et al., 2023). Both biotic and abiotic factors have been proven to influence the ability of crop plants to uptake and accumulate ECs. Biotic factors include plants’ genotype, physiological state, and soil fauna, while abiotic factors include the chemical properties of ECs and environmental perturbations. According to existing relevant studies, the ability of crop plants to uptake and accumulate ECs decreases in the order of leafy vegetables, root vegetables, cereals and fodder crops, and fruit vegetables. However, further studies on crop species are necessary, as ECs have the potential to disturb plant physiological and molecular functions and significantly decrease crop productivity. The presence of ECs in soil-plant systems presents a challenge to agricultural sector. To address the challenges posed by ECs in agriculture, ongoing research, monitoring, and regulatory efforts are essential.

The Research Topic “Emerging contaminants and their effect on agricultural crops” incorporated six original research articles. This Research Topic brought forward the important findings about emerging contaminants of global importance, climatic factors and their impact on plants and environment. Sustainable agricultural practices, improved waste management, and responsible chemical use are also key strategies to mitigate the risks associated with these contaminants. The major outcomes of the studies covered are outlined below.

## Genotype and climatic factors impact on rhizosphere soil properties of Salvia miltiorrhiza

Frequently changing global climatic conditions with serious fluctuations in temperature regimes (high, low or very low temperature), uneven precipitation patterns leading to flooding or drought conditions, high humidity, significant shift in sunshine duration in winters and summers significantly regulate the plant performance in general with heavy yield penalties and specifically reduce the active ingredients and chemical composition in medicinal plants (Zobayed et al., 2005; Manish, 2022; Patni et al., 2022; Appiah et al., 2023; Shaban et al., 2023; Shrestha et al., 2023; Theodoridis et al., 2023). The medicinal plant, Salvia miltiorrhiza Bunge also known as Danshen, belongs to the plant family Lamiaceae bears medicinal properties in traditional Chinese system of medicine to cure Alzheimer’s disease, myocardial infarction, and heart diseases including cerebrovascular diseases in various parts of China (Miroddi et al., 2014; Ma et al., 2017; Cao et al., 2018; Ren et al., 2019; Guo et al., 2020; Zhou et al., 2021; Li et al., 2022). The root extract of Danshen contains chemical constituents which can be divided into liposoluble tanshinones (tanshinone I, tanshinone IIA (TsIIA), tanshinone IIB, cryptotanshinone, and dihydrotanshinone I) and water-soluble phenolics (salvianolic acids) (Ma et al., 2015; Deng et al., 2019; Shi et al., 2019; Sun et al., 2019), Therefore, it becomes imperative to investigate and understand the response of different genotypes of Danshen under different climatic regimes.

He et al. conducted field experiments to evaluate the effect of six genotypes and climatic factors on the biomass and morphological attributes, level of active ingredients, physico-chemical properties of rhizospheric soil, and microbial composition of S. miltiorrhiza at five different cultivation locations Beijing, Anhui, Shandong, Shaanxi and Sichuan, respectively. It has been noted that among six genotypes of Danshen, DS993 proved most suitable genotype for production in terms of growth attributes and active constituents in all the five locations, whereas, DS993 and DS2000 showed promising results for cultivation in Shandong province. Moreover, DS996 came out as unsuitable for cultivation in any of the cultivation sites. The outcome of their investigation allows the farmers to select most suitable genotype for cultivation in the said locations for better productivity and quality (doi: 10.3389/fpls.2023.1110860).

## Nitrogen and phosphorus nutrient cycling in temperate meadow grasslands

Nitrogen (N) is an essential element which is taken up by plants in the form of nitrate (NO_3_^-^) and/or ammonium (NH_4_^+^) and plays crucial role in various plant processes and used for the biosynthesis of important molecules like signal molecules, lipids, photosynthetic pigments, secondary metabolites, photosynthesis and for the formation of proteins and nucleic acids (Guan, 2017; Lee et al., 2020; Musacchio et al., 2020; Liu et al., 2022). Likewise, phosphorus (P) is another vital nutrient usually taken up by the plants as phosphate ions (PO_4_^3-^) and plays a critical role in energy transfer, cell division, and various metabolic processes (Du et al., 2020; Lambers, 2022; Khan et al., 2023; Kumar et al., 2023; McDowell et al., 2023). Both N and P are limiting factors for terrestrial ecosystems and significantly affect the growth and development of plants. Due to various anthropogenic activities and luxurious use of N fertilizers in modern agriculture practices raised the concern due to N deposition in the ecosystems leading to serious ecological consequences (Sun et al., 2022). Excess N deposition in ecosystems in quantities that exceed the N demand of plants leads to a phenomenon known as N saturation which potentially disrupts the nutrient cycling particularly affect the N and P balance in ecosystems. Rather, excessive N can lead to a relative deficiency of P, which may limit plant growth despite an abundance of N. Both N and P biogeochemical dynamics are significantly important for the regulation of terrestrial ecosystems (De Sisto et al., 2023).

Zhang et al. investigated the importance and mechanism of N and P nutrient cycling leading to N deposition in temperate meadow grasslands at Erguna forest-steppe ecoregion research station, Hulunbeier and reported that deposition of N leads to significant change in the structure and function of temperate meadow steppe in terms of nutrient uptake and re-sorption, decomposition of litter and, therefore, significantly affect the nutrient cycling and biogeochemical cycle. They concluded that N deposition significantly modulate the plant nutrient cycling which affects the structure and function of grassland ecosystem (doi: 10.3389/fpls.2023.1140080).

## SiK^®^ fertilization mediated drought resilience of chestnut

Silicon (Si) is the second-most abundant element in the Earth’s crust, and naturally present in soil (Pooniyan et al., 2023). Si primarily accumulates and strengthens the plant cell walls, thus make them more resilient to various abiotic and biotic stresses (Arif et al., 2021; Das et al., 2021; Ranjan et al., 2021; Wang et al., 2021; Ahsan et al., 2023; Manivannan et al., 2023). It has also been reported that Si fertilization potentially enhance the ability of plants to take up other essential nutrients, like Ca and Mg (Katz et al., 2021; Pavlovic et al., 2021; Barão, 2023). Additionally, Si is also important for nutrient cycling in soil and plants (Schaller and Struyf, 2013; Marxen et al., 2016; Li et al., 2018; Xu et al., 2020; Raza et al., 2023).

Carneiro-Carvalho et al. experimented to understand the effect of different concentrations of potassium silicate (0, 5, 7.5, and 10 mM of SiK^®^) fertilization by direct soil application and on the leaves to analyze the biochemical defense response of drought stress -exposed *Castanea sativa* plants in a field experiment in Folhadela belonging to the University of Trás-os-Montes and Alto Douro, Vila Real. It has been noted that SiK^®^ application enhanced the synthesis of soluble proteins and reduced the proline content and markers of oxidative stress such as electrolyte leakage, malondialdehyde and, H_2_O_2_ content plant in tissues, along with the significant increase in the activities of antioxidant enzymes catalase (CAT), ascorbate peroxidase (APX), and peroxidase (POD) activity. Additionally, significant improvement in total phenol compounds and the number of thiols under drought conditions in *Castanea sativa* plants have also been observed. It has been concluded that external application of Si on Castanea sativa plants exerts protective role under water deficit conditions and imparts stress resilience (doi: 10.3389/fpls.2023.1120226).

## Uptake, accumulation and translocation of emerging contaminants in tomato

Ever increasing global population, frequently changing global climatic conditions, land use change and degradation, depleted water sources, COVID outbreak, war and war like situations have intensified significant pressure on food production, and pose serious threat to food security and force to identify and implement sustainable agriculture practices (Steffen et al., 2015; Tully et al., 2019: Tosi et al., 2022; Yang et al., 2023). Irrigation of crop plants limiting resource for crop productivity worldwide and change in land use and deforestration pose extra burden on freshwater resources (Nejadhashemi et al., 2012; Woznicki et al., 2015). Therefore, it is imperative to adapt sustainable agricultural practices to survive global climate change and food security (Rosa, 2022). According to Ungureanu et al. (2020), reclaimed water especially from municipal sources is frequently being used in cropping system of arid and semi-arid regions for crop production. It has also been reported that ~ 44 Nations use treated wastewater for irrigation of agricultural crops (Hashem and Qi, 2021). However, treated wastewater of municipal origin can be an effective and sustainable practice in certain specific circumstances, but it also comes with potential challenges and considerations like salinization of agricultural soil and water, health risks to humans due the presence of pathogens and heavy metals/metalloids (Rogowska et al., 2020; Ben Mordechay et al., 2021; Ben Mordechay et al., 2022; Shi et al., 2022). Recently, ECs has gained the attention due to health and environmental concerns (Taheran et al., 2018).

Denora et al. conducted a pilot-scale study in a field experiment to understand the uptake, accumulation and translocation of ECs in tomato (*Solanum lycopersicum* L. cv Taylor F1) plants irrigated with tertiary treated wastewater effluent in Southern Italy and noted that in TWWx3 strategy, clarithromycin, carbamazepine, metoprolol, fluconazole, and climbazole exhibited interactions with the soil-plant system, with varying degradation rates, soil accumulation rates, and plant accumulation rates, whereas, naproxen, ketoprofen, diclofenac, sulfamethoxazole, and trimethoprim showed degradation. They concluded that Fluconazole and carbamazepine have high plant absorption concentrations, with accumulation evident in the leaves, roots, and berries of the TWWx3 treatment (doi: 10.3389/fpls.2023.1238163).

## Soil polyethylene/polyvinyl chloride microplastics impact on soybean

Synthetic non-biodegradable plastic products made up of polyethylene (PE), polyvinylchloride (PVC), nylon, polypropylene, polystyrene, and polylactic acid in ecosystem degrade into microplastics (MPs) and even into nano-plastic pose serious threat to aquatic and terrestrial ecosystems (Duis and Coors, 2016; Hale et al., 2020; Hamidian et al., 2021; Akdemir and Gedik, 2023; Chen et al., 2023; Akdemir and Gedik, 2023). The impact of soil microplastics (MPs), particularly polyethylene (PE) and polyvinyl chloride (PVC) as ECs attracted the global attention in terms of environmental and agricultural challenges (Walker and Fequet, 2023; Cha et al., 2023; Jimoh et al., 2023). MPs coined by Thompson et al. (2004) are tiny plastic particles (<5 mm), and can originate from a variety of sources, such as the breakdown of larger plastic items, microbeads in personal care products, agricultural plastic films (plastic mulch), use of treated wastewater of municipal origin for irrigation and are present in most of the ecosystems as well as in biological samples (Leslie et al., 2022; Osman et al., 2023). MPs accumulation in the soil disturb the soil physical properties such as bulk density, water holding capacity, soil fertility, and soil ecosystem (Rillig, 2012; de Souza MaChado et al., 2018; Kublik et al., 2022). Factors such as the type and size of microplastics, soil conditions, and crop species all play a role in determining the extent of the impact.

Li et al. studied the impact of emerging pollutants MPs such as PVC and PE alone and in combination on the photosynthetic performance of soybean (*Glycine max* [L.] merr.) (c.v. Zhonghuang 37) plants grown in the experimental field of experiment station of shandong agricultural university. It has been noted that MPs–PVC and –PE alone and in combination increased the lipid peroxidation by 21.8-97.9%, whereas, a significant decrease in photosynthetic efficiency (net photosynthetic rate, PSII activity, energy uptake, capture, transport, and dissipation) by 11.5-22.4% has also been observed when compared with the control plants. It has also been reported that PVC alone showed a greater negative impact on soybean plants in comparison to PE single stress and control plants (doi: 10.3389/fpls.2022.1100291).

## Effect of nano-titanium dioxide on mulberry: transcriptomics and metabolomics perspective

Nanomaterials and nanoparticles (NPs) have attracted the global attention for their widespread application in almost all sectors including modern agricultural practices (Zain et al., 2022; Chauhan and Gill, 2023). Nano-agrochemicals have vast potential in complementing the growth, development and yield of crop plants along with resilience to variety of biotic and abiotic stress factors (Liliane and Charles, 2020; Shahzad et al., 2021; Beig et al., 2022; Upadhayay et al., 2023). Among NPs, titanium dioxide nanoparticles (TiO_2_ NPs) are reported to be widely used NPs in paints, sunscreen, paper, antibacterial fiber, fine ceramics, plastic, food additives due to their anti-UV, antibacterial, self-cleaning, anti-aging, anti-fading, chemical inertness, photocatalytic activity (Hendren et al., 2011; Kovačič et al., 2022). Mixed observations have been reported by the researchers on the effect (synergistic or antagonistic) of TiO_2_ NPs on various crop plants (Bueno et al., 2022).

Yu et al. investigated the effect of different concentrations of TiO_2_ NPs (0, 100, 200, 400 or 800 mgl^-1^) on the growth performance of mulberry (*Morus Alba* L.) seedlings employing transcriptomics and metabolomics approaches. The authors noted that the TiO_2_ NPs are easily taken up by the mulberry roots and transferred to the above-ground parts which results in the destruction of root and shoot system of mulberry plants. Additionally, TiO_2_ NPs application reduced the chloroplast number and pigment concentration and drastically increased the rate of lipid peroxidation measured in terms of malondialdehyde content. Furthermore, the results of transcriptomic data revealed that TiO_2_ NPs application significantly affected the expression of genes involved in protein metabolism, energy synthesis and transport, and stress responsive genes. Moreover, metabolomic study pointed out significant difference in 42 metabolites in mulberry. Out of 42 metabolites, 26 showed up-regulation in expression, whereas, the rest of the 16 metabolites were noted to be down-regulated. The down-regulated metabolic pathways belong to the TCA cycle, citric acid cycle, and, secondary metabolite biosynthesis pathway which was not suitable for the germination and/or growth of the mulberry saplings. The research concluded that the application or exposure of plants to TiO_2_ NPs should be comprehensively monitored and evaluated to avoid potential risks of NPs on plants (doi: 10.3389/fpls.2023.1175012).

## Conclusions and outlook

ECs are fascinating to the global public, research, and policy attention owing to their potential health risks. Despite the tremendous progress made in this field, the occurrence, sources, and hazards of ECs have not been fully understood and full-scale implementation of effective technologies to meet practical application requirements has not been achieved. To address these challenges, it is essential to focus research efforts on robust remediation approaches developed on sustainable foundations. These approaches should aim to establish consistency between the characteristics of pollutants and the integration of multiple removal processes to ensure compliance with regulations. A state-of-the-art review encompassing various studies on ECs and the strategies adopted by both developed and developing countries to combat their release is crucial. This review should consider factors related to water sustainability, such as water availability, usage patterns, generation, pollution management, and the health of aquatic systems. There is an urgent need to develop standards and policy strategies regarding limits of the ECs concentrations to assess the environmental and potential human exposure risks of ECs originating from various sources.

## Author contributions

MN: Conceptualization, Writing – original draft, Writing – review & editing. RG: Conceptualization, Writing – original draft, Writing – review & editing. SG: Conceptualization, Validation, Writing – original draft, Writing – review & editing. KS: Conceptualization, Writing – review & editing. AS: Conceptualization, Writing – review & editing. NT: Conceptualization, Writing – original draft, Writing – review & editing.
